# Unusual case of severe fever with thrombocytopenia syndrome showing clinical manifestations in a companion dog

**DOI:** 10.1002/vms3.261

**Published:** 2020-04-12

**Authors:** So‐Jeong Nam, Ye‐In Oh, Hyun‐Jung Kim, Doo‐Sung Cheon, Sung‐Jun Noh, Yeon‐Jung Hong

**Affiliations:** ^1^ Western Animal Medical Center Seoul South Korea; ^2^ Chungnam National University Daejeon South Korea; ^3^ Animal and Plant Quarantine Agency Gimcheon South Korea; ^4^ Postbio Inc. Guri‐si South Korea; ^5^ Naeum Animal Medical Center Cheongju South Korea

**Keywords:** dog, Huaiyangshan banyangvirus, Republic of Korea, severe fever with thrombocytopenia syndrome, tick

## Abstract

Severe fever with thrombocytopenia syndrome (SFTS) virus is an emerging zoonotic virus in East Asia. However, SFTS virus (SFTSV) has not been reported to cause clinical infection in companion dogs to date. We report the case of a 4‐year‐old companion dog that presented with fever, vomiting, leukocytopenia and thrombocytopenia at a veterinary hospital in the Republic of Korea. It was diagnosed with SFTS, which was confirmed using real‐time reverse transcription PCR, sequencing and an indirect immunofluorescence assay, and recovered after supportive care. Further studies are required to investigate SFTSV infection in companion animals, living in close contact with humans, as well as animal‐to‐human transmission.

## Graphical abstract

A 4‐year‐old companion dog presented with fever, vomiting, leukocytopenia, and thrombocytopenia at a veterinary hospital in Republic of Korea. It was diagnosed with severe fever with thrombocytopenia syndrome (SFTS) and recovered after supportive care. To our knowledge, this is the first report of SFTSV infection with clinical manifestations in a companion dog.

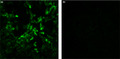

## 1 INTRODUCTION

Severe fever with thrombocytopenia syndrome (SFTS) is an emerging tick‐borne zoonotic disease in East Asia, including the Republic of Korea, China, Japan and Vietnam (Chen et al., 2019; Tran et al., 2019), and has a high mortality rate of 16.2%–55% in humans (Im et al., 2019; Li et al., 2018; Takahashi et al., 2014; Yu et al., 2011; Zhang et al., 2012) and of 62.5% in cats (Matsuu et al., 2019). The tick *Haemaphysalis longicornis* is the main vector of the SFTS virus (SFTSV; Huaiyangshan banyangvirus: Phenuiviridae), previously described as Phlebovirus (Maes et al., 2019), and is the most dominant tick species (88.9%) in the Republic of Korea (Kim et al., 2015). Although antibodies to SFTSV have been detected in various animal species, with a seroprevalence ranging from 0.2% to 52.1% in dogs, most animals are asymptomatic (Chen et al., 2019; Ding et al., 2014; Kang et al., 2019; Lee et al., 2017, 2018; Oh et al., 2016). However, in stray and pet cats, there have been reports of apparent SFTSV infections manifesting with clinical symptoms (Kida et al., 2019; Matsuu et al., 2019), and there was a report of transmission of SFTSV from these cats to the attending veterinarian (Kida et al., 2019).

To our knowledge, there have been no reports of apparent SFTSV infection with clinical symptoms in companion dogs. Here, we describe the progress in diagnosis of an unusual case of clinical SFTSV infection that developed after a tick bite in a companion dog in the Republic of Korea.

## 2 MATERIALS AND METHODS

### 2.1 Clinical history of the canine patient

The dog, a 4‐year‐old, castrated male, Bichon Frise, presented with a 2‐day history of fever, anorexia, lethargy and vomiting in October 2018 (day 0: first day of admission). The owner had noticed multiple tick bites and applied a tick‐preventative medicine 19 days prior to admission. Physical examination and a complete blood count revealed fever (40.6°C; reference, 38.0°C–39.2°C), leukopenia and thrombocytopenia without bleeding (Table 1). On day 2, diagnostic imaging revealed slight enlargement of the spleen and pancreas and a thickened gall bladder wall. Real‐time polymerase chain reaction (PCR) for common infectious diseases in dogs (*Anaplasma* spp., *Ehrlichia* spp., *Babesia* spp., *Leptospira* spp., *Bartonella* spp., haemotropic mycoplasma and *Rickettsia* spp.) showed negative results. We suspected SFTSV infection on the basis of the history, fever and bicytopenia without anaemia.

**TABLE 1:** Laboratory parameters during the course of hospitalization for the companion dog with severe fever with thrombocytopenia syndrome

		Day							
Parameters	Reference range	0	1	2	3	5	7	9	11
White blood cell	5.2–13.9 K/µl	2.3	1.7	1.25	6.3	12.48	7.23	7.21	6.41
Neutrophil	3.9–8 K/µl	1.2	1.3	0.73	3.93	8.8	4.76	4.51	4.15
Lymphocyte	1.3–4.1 K/µl	1	0.3	0.37	1.65	2.27	1.62	1.93	1.73
Monocyte	0.2–4.1 K/µl	0.1	0.1	0.06	0.33	1.15	0.66	0.58	0.42
Platelet	143.3–400 K/µl	44	25	29	44	55	48	85	164
C‐reactive protein	0–20 mg/dl	153	138	87.6	65	43.6	32.7	12.7	<10
ALT^a^	19–70 U/L	NE^d^	185	157	NE	62	NE	NE	27
AST^b^	15–43 U/L	NE	228	333	NE	75	NE	NE	19
ALP^c^	15–127 U/L	NE	1,512	353	NE	407	NE	NE	184
D‐dimer	0–0.3 µg/ml	NE	NE	1.6	NE	0.8	NE	NE	<0.1

Note

Day 0 means the first day of admission to a veterinary hospital.

^a^ Alanine aminotransferase.

^b^ Aspartate aminotransferase.

^c^ Alkaline phosphatase.

^d^ Not examined.

To confirm SFTSV infection, we acquired a whole blood sample and performed real‐time reverse transcription (RT)‐PCR for specific identification of the S‐segment genome, a Vero E6 cell culture for virus isolation, and an indirect fluorescence assay (IFA) (day 2). To determine the genetic identity of the virus, we amplified the full length of the S‐segment of SFTSV.

### 2.2 Detection of SFTSV RNA in canine serum samples

SFTSV RNA was extracted from the patient's serum samples using an RNAeasy extraction kit (QIAgen) with an automated extraction system (QIAcube, QIAgen). The eluted RNA was added to a reaction mixture provided in the RT‐PCR kit (Bioneer). SFTSV‐specific TaqMan primers and probe were used to detect viral RNA reliably. The primers (5′‐GGGTCCCTGAAGGAGTTGTAAA‐3′ and 5′‐TGCCTTCACCAAGACTATCAATGT‐3′) and probe (5′‐FAMTTCTGTCTTGCTGGCTCCGCGC‐TAMRA‐3′) were designed to target conserved nucleic acid sequences (Cui et al., 2012). For RT‐PCR, the amplification profile consisted of the following steps: 50°C for 15 min, 95°C for 5 min, 95°C for 30 s and 60°C for 30 s, for 45 cycles, using an RGQ qPCR machine (QIAgen). The cut‐off of cycle threshold (Ct) value for a positive reaction was set to 35 cycles.

### 2.3 Quantification of RNA of SFTSV

To measure viral loads of SFTSV in the dog's serum samples, qRT‐PCR was performed using TaqMan probes. Based on a standard curve produced using the Ct values of a plasmid containing the S segment, the SFTSV RNA copy number (per ml) was determined.

### 2.4 Virus isolation and identification of SFTSV by IFA

Canine serum samples collected from the patient were inoculated onto monolayers of Vero E6 cells for virus isolation, as previously described (Lee et al., 2016; Yu et al., 2011). After adaptation and proliferation of viruses in Vero E6 cells, we confirmed SFTSV replication by IFA using anti‐SFTSV rabbit polyclonal antibody, as described (Qu et al., 2012). Because of the infectivity of SFTSV, all experiments were conducted in a biosafety level 3 facility in Korean Animal and Plant Quarantine Agency, according to the institutional biosafety operating regulations and procedures.

### 2.5 Phylogenetic analysis of SFTSV

The S segment of SFTSV RNA was amplified using one‐step RT‐PCR, as previously described (Hwang et al., 2017; Li et al., 2018). Positive samples of RT‐PCR were sequenced using the Sanger sequencing technique. Sequence alignment was computed using the software program (DNAstar). The phylogenetic trees were analysed using the Clustal W method in the DNAstar package.

## 3 RESULTS

Based on clinical characteristics and test results, we diagnosed the dog with SFTS and secondary acute pancreatitis. Because there is no established treatment for SFTS in dogs, we provided supportive care, including fluid therapy, analgesics, antibiotics, an antiemetic and an antacid, with continuous monitoring. The dog showed a normalized white blood cell count and decreased C‐reactive protein level, with no fever or vomiting, on day 3. Clinical signs, including anorexia and lethargy, were completely resolved by day 9, with remarkable improvement in the platelet count. On day 11, viral RNA (1.3 × 10^4^ copies/ml) was still detected despite normal platelet counts and biochemistry findings. On day 13, the results of real‐time RT‐PCR were negative, and the viral load was below the detection limit; therefore, the patient was discharged.

**FIGURE 1:**
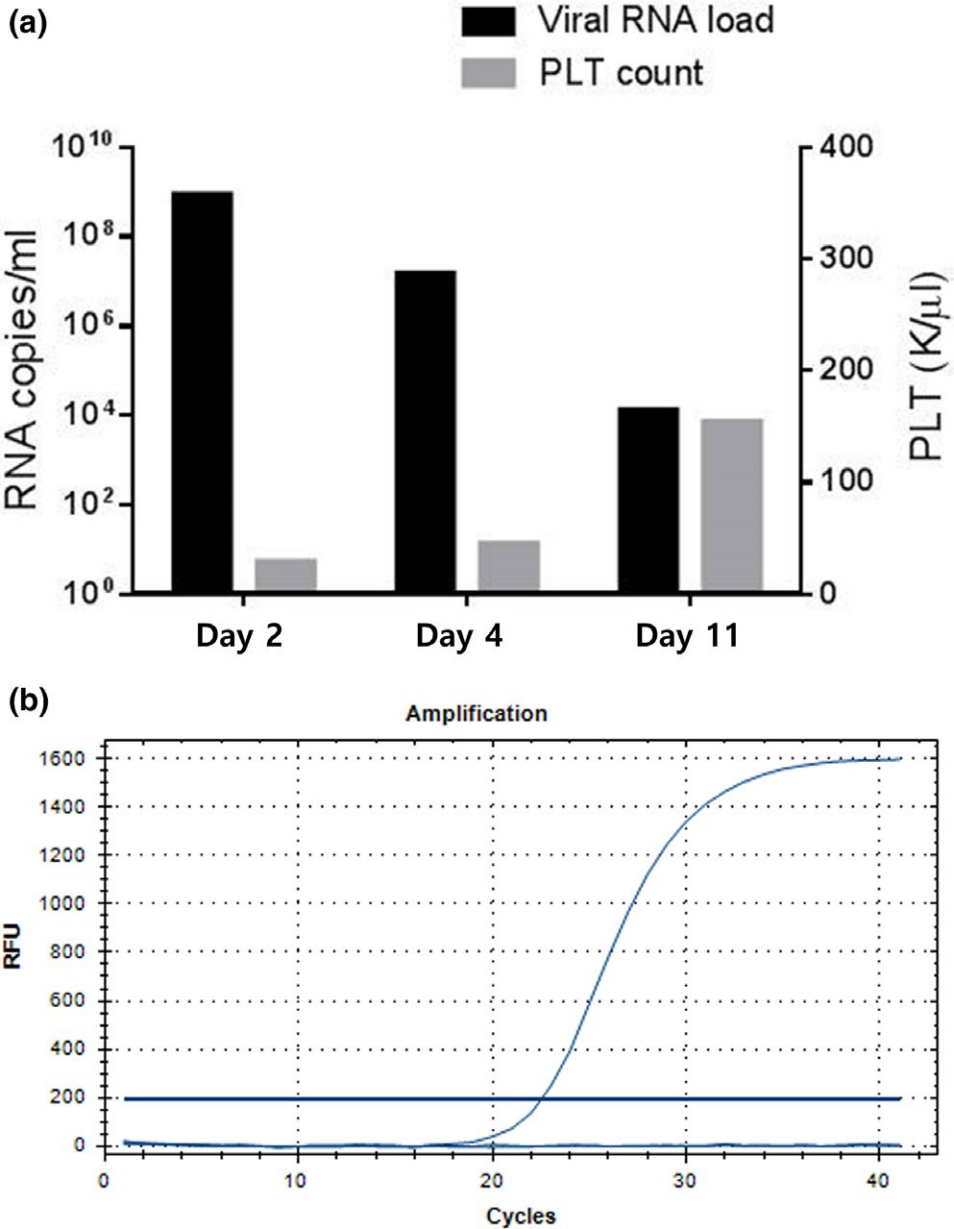
Findings of consecutive reverse‐transcription polymerase chain reaction (PCR) for the monitoring of viral RNA loads in a companion dog with severe fever with thrombocytopenia syndrome (SFTS). (a) The image shows that the viral RNA load gradually decreased while the platelet count increased over time. (b) Quantitative (q)PCR amplification plot was produced using SFTS virus (SFTSV)‐specific primers and a Taqman probe set. We determined positivity based on Ct values determined automatically by qPCR program. The cut‐off was under the 35 Ct value. The clinical specimen was confirmed as positive by SFTSV Taqman‐based RT‐qPCR, with a Ct value 23.5

**FIGURE 2:**
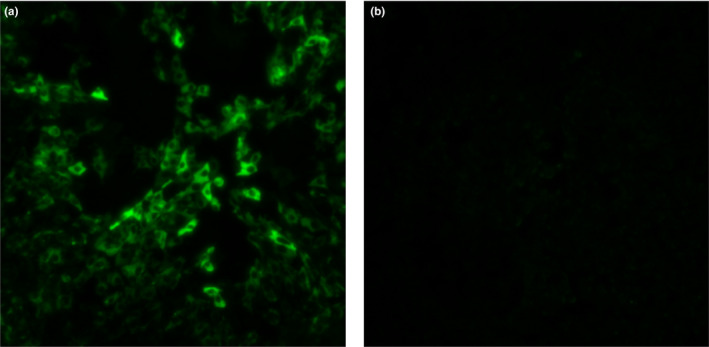
Results of virus isolation in a Vero E6 cell culture based on a blood sample obtained on day 2 of hospitalization of the companion dog with severe fever with thrombocytopenia syndrome (SFTS). (a) A sample of the canine patient with SFTSV. (b) A sample with mock infection

**FIGURE 3:**
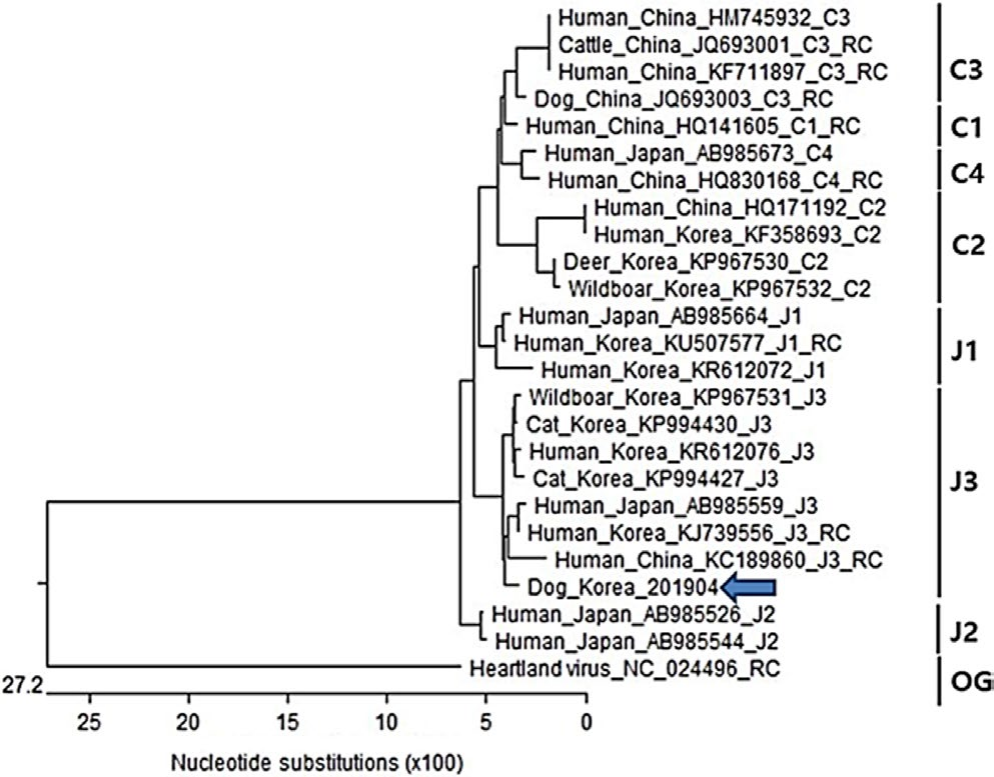
Phylogenetic analysis of severe fever with thrombocytopenia syndrome virus (SFTSV) based on the S‐segment of the virus. The name of sequence files was described by the host species, countries, GenBank accession numbers. The sequences identified in the present study are indicated by arrows. The partial sequences of s segment of heartland virus were used for outgroup (OG). The clades are designated as described by Yoshikawa et al. (2015)

Real‐time PCR facilitated the detection of viral RNA up to day 13; the viral RNA load on day 2 was 8.5 × 10^8^ copies/ml (Figure 1). The Vero E6 cell culture and IFA confirmed the presence of live viruses as causative agents of the fever and thrombocytopenia (Figure 2). The partial S segment of SFTSV was confirmed from the patient's sample by real‐time RT‐PCR and RT‐PCR. Phylogenetic analysis of partial S fragment sequences (346 bp) revealed a sequence that was highly conserved in the Bunyaviridae. The resulting phylogenetic tree of the S segment showed that the isolate from the patient was similar to SFTSV strains isolated from humans in the Republic of Korea detected (98.01% similarity) (GenBank accession no. KR612079.1), which belonged to clustering genotype J3 (Liu et al., 2012) (Figure 3).

## 4 DISCUSSION

Although SFTSV is primarily transmitted through tick bites, human‐to‐human and cat‐to‐human transmission, nosocomial infection are also possible (Kida et al., 2019; Matsuno et al., 2018; Shimazu, Saito, Kobayashi, Kubo, & Nohgawa, 2018). The pooled serum prevalence in various animal species is 0.33%–100% (Chen et al., 2019); moreover, rearing animals is a risk factor for human SFTS (Song, Lee, & Ju, 2017). Consequently, SFTS is attracting attention as an emerging zoonotic disease. In particular, companion animals, such as dogs and cats, which live in close contact with humans, serve as important mediators for transmitting infectious diseases to humans. Therefore, it is important to investigate infectious diseases in companion animals to gain information related to preventing infection in humans. In the present case, the dog's owner showed no clinical signs of SFTS and did not undergo the required examinations; hence, we could not identify any association. However, SFTSV isolated from the dog strongly resembled that previously isolated from humans in the Republic of Korea (Liu et al., 2012); therefore, we cannot negate the possibility of dog‐to‐human transmission. Further studies are necessary, given that cat‐to‐human transmission has been proven previously (Kida et al., 2019).

Human SFTS presents various clinical manifestations, including high fever, gastrointestinal signs, neurological signs, thrombocytopenia, leukocytopenia, lymphadenopathy and multiple organ failure (Sun et al., 2012). The symptoms observed in our case also included fever, vomiting, leukocytopenia and thrombocytopenia. Although a high viral load is a strong risk factor for death in humans (Sun et al., 2012), human risk factors cannot be applied to dogs. Our patient exhibited extremely high viraemia (≥10^8^ copies/ml); however, when the viral load was reduced, clinical signs improved, eventually resulting in recovery and survival. Considering the variability in risk factors for SFTS‐associated death, it was difficult to assess the prognosis in this case. In addition, less‐severe forms of SFTS have been reported in humans, and thus, the possibility of less‐severe disease in animals should be considered (Sun et al., 2015).

In animals, the clinical manifestations of SFTS have only been reported in cheetahs and cats (Matsuno et al., 2018; Matsuu et al., 2019; Park et al., 2019). To our knowledge, despite the high pooled seroprevalence (29.5%) of SFTSV antibody in dogs (Chen et al., 2019), there are no reports of confirmed SFTSV infection in companion dogs with suspected SFTS, showing clinical signs. Because of limited literature on animal SFTS, which is still an emerging infectious disease, veterinarians are unaware of this possibility and may overlook this as a diagnosis. Considering the time required for host adaptation, the incidence of SFTS symptoms may increase in animals in future.

In conclusion, SFTS exhibiting clinical manifestations in a companion dog has not been reported to date, to the best of our knowledge. SFTS is an emerging zoonotic disease with an annual increase in incidence (Im et al., 2019); thus, thorough tick prevention measures for companion dogs and measures for preventing animal‐to‐human transmission are necessary.

## CONFLICT OF INTEREST

The authors have no conflict of interests.

## AUTHOR CONTRIBUTION

**So‐Jeong Nam:** Conceptualization; Data curation; Formal analysis; Investigation; Methodology; Resources; Visualization; Writing‐original draft. **Ye‐In Oh:** Conceptualization; Data curation; Formal analysis; Investigation; Methodology; Project administration; Resources; Software; Supervision; Validation; Visualization; Writing‐original draft; Writing‐review & editing. **Hyun‐Jung Kim:** Conceptualization; Data curation; Formal analysis; Investigation; Methodology; Supervision; Validation; Visualization. **Doo‐Sung Cheon:** Conceptualization; Data curation; Formal analysis; Funding acquisition; Investigation; Methodology; Resources; Software; Supervision; Validation; Visualization; Writing‐review & editing. **Sung‐Jun Noh:** Data curation; Formal analysis; Investigation; Methodology; Resources; Supervision. **Yeon‐Jung Hong:** Conceptualization; Data curation; Formal analysis; Investigation; Methodology; Project administration; Resources; Supervision; Validation; Writing‐review & editing.

## ETHICAL STATEMENT

The authors confirm that the ethical policies of the journal, as noted on the journal's author guidelines page, have been adhered to. No ethical approval was required as this is a description of the diagnosis and treatment of one case and no experimentation was conducted on the treated dog.
